# Beyond Skin Rash: Alpelisib-Induced Anaphylactic Reactions

**DOI:** 10.1093/oncolo/oyad092

**Published:** 2023-04-22

**Authors:** Tim Schutte, Laurien J Zeverijn, Birgit S Geurts, Gijsbrecht F de Wit, Marleen Kok, Frans L Opdam

**Affiliations:** Amsterdam UMC location Vrije Universiteit Amsterdam, Department of Medical Oncology, de Boelelaan, Amsterdam, The Netherlands; The Netherlands Cancer Institute, Division of Molecular Oncology and Immunology, CX Amsterdam, The Netherlands; The Netherlands Cancer Institute, Division of Molecular Oncology and Immunology, CX Amsterdam, The Netherlands; The Netherlands Cancer Institute, Division of Molecular Oncology and Immunology, CX Amsterdam, The Netherlands; The Netherlands Cancer Institute, Department of Medical Oncology, CX Amsterdam, The Netherlands; The Netherlands Cancer Institute, Department of Medical Oncology, CX Amsterdam, The Netherlands

## Abstract

Alpelisib is a specific oral PI3K inhibitor used combined with fulvestrant for the treatment of patients with HR+/HER2–/*PIK3CA-*mutated metastatic breast cancer. Adverse drug reactions with alpelisib are common, including hyperglycemia and rash. Here we describe extraordinary and life-threatening reactions beyond skin rash in two patients with progressive *PIK3CA-*mutated metastatic cancer in whom alpelisib was initiated. Case-A (vaginal cancer): After 10 days on treatment, she developed dry eyes, generalized rash and itching. Alpelisib was interrupted and symptomatic treatment initiated. Because of an initial tumor response, a rechallenge was done. Ninety minutes after a reduced dose of alpelisib, she developed an anaphylactic reaction with angioedema, hypotension, and skin rash. Case-B (breast cancer): After 11 days on treatment, she developed skin rash and alpelisib was interrupted. At re-initiation, she felt tingles in her face and ears and some skin erythema. Given the mild rash, a second rechallenge with premedication was performed. Ninety minutes after a reduced dose of alpelisib, she developed a type-1 allergic reaction with angioedema, tingles, and skin rash. In both cases, a type-1 allergic reaction was diagnosed and symptomatic treatment was initiated, alpelisib was permanently discontinued and the patients fully recovered the next week(s). This report underlines the critical importance to consider type-I allergic reactions in the differential diagnosis in cases of rash associated with alpelisib. Even if a reaction develops after days on treatment, a type-I allergic reaction cannot be excluded. A rechallenge can be dangerous and should always be well contemplated or even avoided.

## Teaser

Alpelisib combined with fulvestrant is used to treat patients with HR+/HER2–/PIK3CA-mutated metastatic breast cancer. Adverse drug reactions with alpelisib are common, including hyperglycemia and rash. This article describes extraordinary and life-threatening reactions beyond skin rash in two patients with progressive PIK3CA-mutated metastatic cancer.

**Implications for Practice**

Adverse drug reactions with alpelisib are common and include hyperglycemia and rash. Additional clinical findings beyond rash, such as urticaria, mild facial angioedema, edema, fever, dyspnea, or tachycardia, should be explicitly evaluated. Even if an initial reaction develops slowly after days, it could still be a (developing) type-I allergic reaction. Especially in patients with symptoms or signs beyond rash, such as urticaria or facial (angio)edema, which are suggestive of a hypersensitivity reaction, a rechallenge could be dangerous and should always be well contemplated or even avoided if the suspicion of a type-I allergic reaction is high.

## Introduction

Alpelisib is a specific oral inhibitor of the enzyme phosphatidylinositol 3-kinase (PI3K) α-isoform and is used in the treatment of locally advanced/metastatic breast cancer. It is the first FDA and EMA approved PI3Kα inhibitor for the treatment of *PIK3CA*-mutated, hormone receptor-positive (HR+), HER2-negative metastatic breast cancer, when given in combination with fulvestrant.^1^ Although effective in a substantial proportion of patients, adverse drug reactions (ADRs) of alpelisib are common, including hyperglycemia and skin rash. Even in case of severe skin rash (CTCAE grade 3, with >30% body surface area involved and active skin toxicity), a rechallenge with lower dose upon resolution of rash grade ≤1 is recommended.^2-4^ Skin rash reactions should be distinguished from hypersensitivity reactions (including anaphylactic reaction) as the alpelisib label recommends permanently discontinuation without eventual re-introduction in patients with serious hypersensitivity reactions. Here we describe two cases of alpelisib rechallenge resulting in type-I allergic/anaphylactic reactions. It is therefore important to recognize alpelisib associated type-I allergic reactions beyond skin rash.

### Case A:

A 54-year-old woman with metastatic vaginal cancer was referred to our center because of progression on previous treatments including carboplatin/paclitaxel, capecitabine, chemo-radiation and nivolumab. In search of actionable tumor mutations, whole-genome sequencing (WGS) was performed, which identified an activating mutation in the *PIK3CA* gene. At present alpelisib is not registered, nor reimbursed for the treatment of tumors other than breast cancer with *PIK3CA* mutations. However, patients could be enrolled in The Drug Rediscovery Protocol (DRUP) trial, a nationwide clinical trial which facilitates the use of approved targeted therapies beyond their original label in cohorts of molecularly similar cancers and evaluates effectiveness and safety of this approach (NCT02925234).

**Figure 1:**
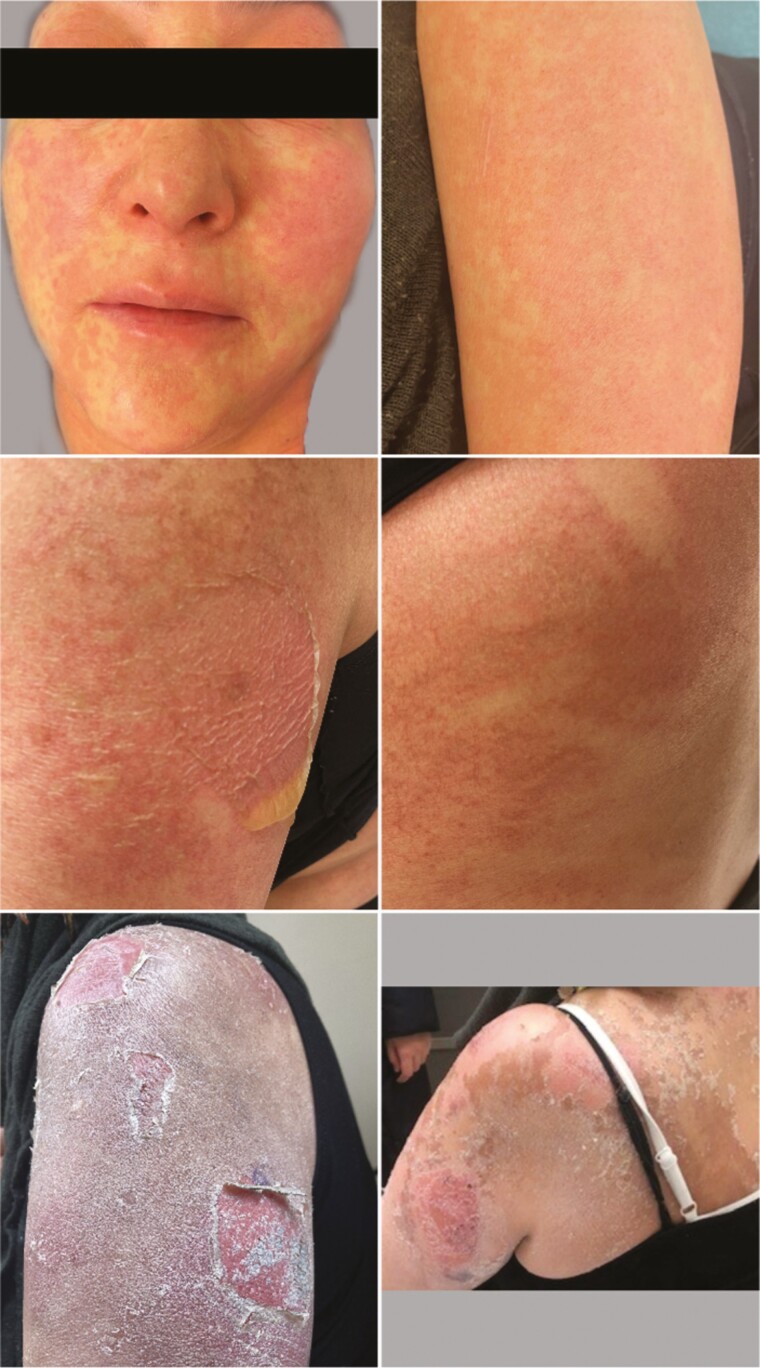
Pictures of the patient described in case A: Upper left panel: erythematous maculopapular rash with (very) mild edema in the face an eyelids (not shown for privacy reasons) (photo taken on day 13 after start of alpelisib). Upper right panel: erythematous maculopapular rash of the left arm (photo taken on day 13). Central panels (day 6 after rechallenge, day 34 after initial start of alpelisib): Bulla of the left arm with erythema (central – left), bulla on back with erythema (central panel – right) and pubis (not shown). Assessment by dermatologist, most likely Bullous toxicoderma. Lower panel left: Peeling skin, or desquamation of left forearm with a white shade as a result of applied zinc ointment (day 6 after rechallenge) Lower panel right: some residue of peeling skin with underneath healed skin (day 14 after rechallenge).

After baseline study screening, treatment with alpelisib 300 mg QD was started (without prophylactic antihistamines). After 10 days of treatment, the patient developed dry eyes, an itching skin and a generalized rash on arms, legs, chest, and back. We advised to interrupt alpelisib and scheduled a visit the next day. On physical examination the patient was hemodynamically stable and her skin was erythematous in a maculopapular pattern covering >30% of her body surface area (BSA), assessed as a grade 3 maculopapular rash (CTCAE v.5) (Figure 1).

Given the extensive reports of alpelisib-induced rash,^2,3,5^ we considered a (regular) toxicodermal skin rash the most likely diagnosis. Still, given the clinical finding of some edema in her face, we also considered an allergic reaction in our differential diagnosis. We treated her symptoms initially with levocetirizine 10 mg QD (histamine receptor antagonist) with lubricating skin and eye ointments to relieve itching and her dry eyes, topical/systemic steroids were considered but omitted. In the days thereafter, her symptoms demised and she soon recovered. Subsequently, we had to decide how to proceed, taking into account that the patient experienced a significant decrease in the vaginal tumor mass, suggesting clinical benefit.

Given the recommendations to rechallenge in case of rash^2,3^ together with the signs of clinical benefit, we discussed the risks and benefits of a continuation/restart with alpelisib with levocetirizine as pre-medication. Given the mild edema during the initial reaction, we proposed an in-hospital observed rechallenge with a reduced dose of alpelisib, together with levocetirizine. The rechallenge was initiated 16 days after the start of the initial reaction, with complete resolution of the edema and rash. Ninety minutes after a reduced dose of alpelisib (150 mg) was administered, the patient developed an anaphylactic reaction with angioedema with (mildly) swollen lips, edema in her face, chills, fever, hypotension, and reappearance of skin rash. No evident airway edema or obstruction was observed. Symptomatic treatment with clemastine, hydrocortisone, and IV fluids for this serious anaphylactic reaction were promptly initiated, adrenaline was considered but not given (mean arterial pressure (MAP) was ≥65 mmHg and no airway edema/obstruction) and alpelisib was permanently discontinued. Mild hypotension persisted for ±48 hours; however, urine production as sign of organ perfusion was sufficient, without significant laboratory abnormalities (Table 1). A skin reaction persisted wherefore levocetirizine and ointments (including topical steroids) were continued. At a follow-up visit 6 days later, two bullae were present (Figure 1) for which the dermatologist was consulted and prednisone 30 mg QD was started. In the days thereafter, no new bullae emerged, the steroids were tapered and stopped and the patient made a full recovery in the following weeks. Although the 8-week evaluation CT-scan showed stable disease, palliative radiotherapy was initiated for local pain of the vaginal mass, indicating local progression.

**Table 1.** Laboratory parameters after clinical rechallenge of both patients.

	Patient A*	Patient B Day 27**	Reference value
Hemoglobin	6.7 mmol/l	8.3 mmol/l	7.5-10.0 mmol/l
Hematocrit	0.32	0.41	0.35-0.45
Erythrocyte count	3.4 × 10^12^/l	3.9 × 10^12^/l	4.0-5.0 × 10^12^/l
M.C.V. (Ht/Rbc)	96 fl	105 fl	80-100 fl
Leukocyte count	7.2 × 10^9^/l	11.4 × 10^9^/l	4.0-10.5 × 10^9^/l
Neutrophil count	6.5 × 10^9^/l	10.7 × 10^9^/l	0.5-7.5 × 10^9^/l
Eosinophilic granulocyte count	< 0.1 × 10^9^/l	0.1 × 10^9^/l	<0.5 × 10^9^/l
Thrombocyte count	178 × 10^9^/l	288 × 10^9^/l	150-400 × 10^9^/l
Creatinine	79 µmol/l	84 µmol/l	
GFR (MDRD-4)	65 ml/min/1.73 m^2^	63 ml/min/1.73 m^2^	
ASAT	87 U/L	28 U/L	<31 U/L
ALAT	78 U/L	32 U/L	<34 U/L
Tryptase	5.8 μg/l	n.a.	<11.4 μg/l

*43hr after clinical rechallenge.

**27 days after initial start, 23:45hr hours after clinical rechallenge.

### Case B:

**Figure 2:**
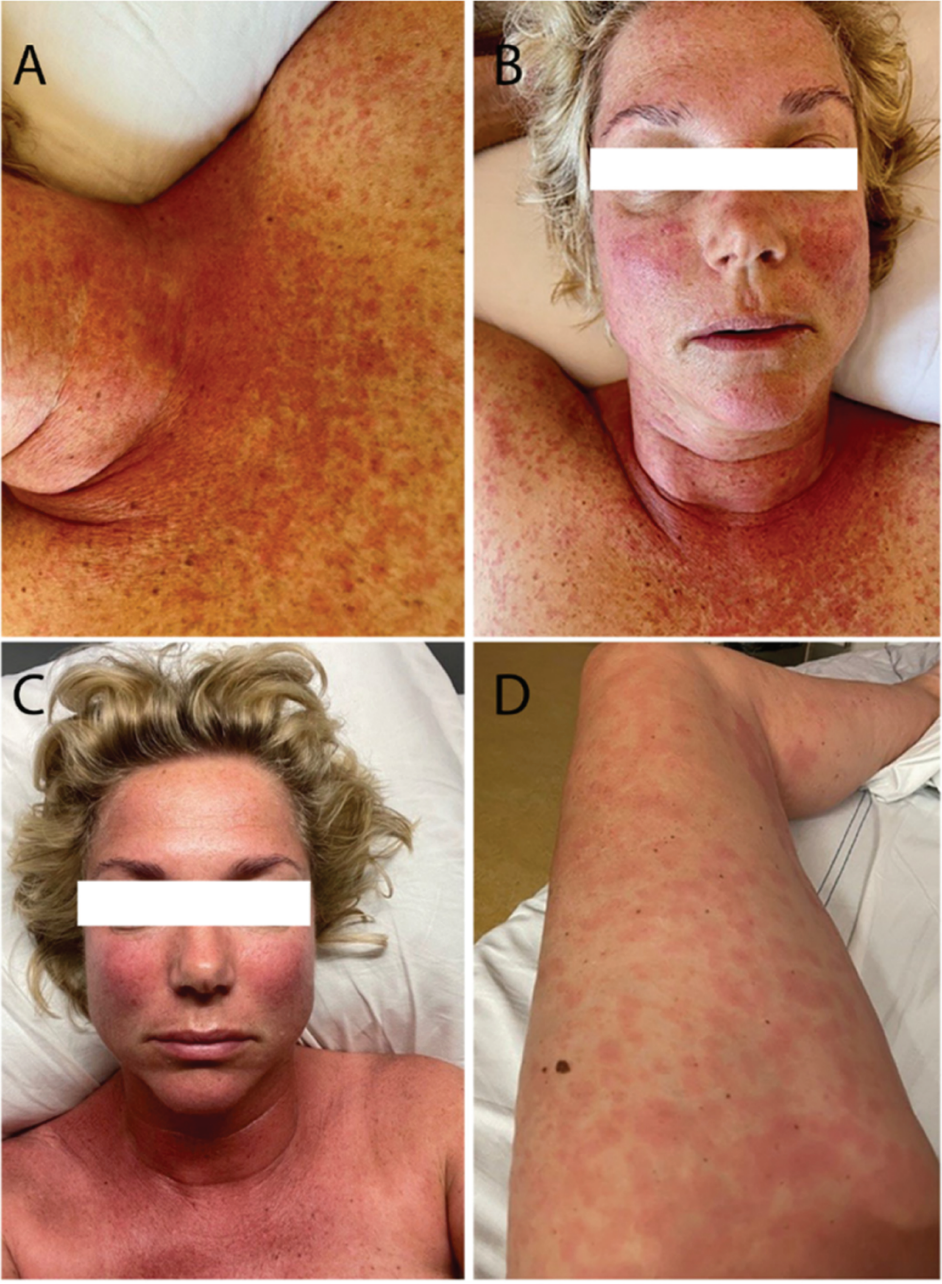
Pictures of the patient described in case B: Panels A and B (9 days after initial start of alpelisib): erythematous maculopapular rash. Panel C: erythematous rash with mild edema in the face an eyelids (eyes not shown for privacy reasons) (photo taken the day after the second (clinical) rechallenge). Panel D: urticaria on left leg (photo taken the day after the second (clinical) rechallenge).

A 49-year-old woman with metastatic breast cancer was referred to our center because of progression on previous treatments including doxorubicin/cyclophosphamide, paclitaxel, anastrozole/gonadoreline, tamoxifen, and palbociclib/fulvestrant. Capecitabine was discontinued due to cardiac spasms and hand-feet-syndrome. Next-generation sequencing (NGS) using a gene-panel had identified activating *PIK3CA* mutations. Alpelisib 300 mg QD (combined with fulvestrant) was initiated and 5 days thereafter, the patient reported elevated fasting glucose levels. She was started on metformin following the SOLAR-1 glucose regulation protocol.^2^ Eleven days after the first administration, the patient developed a skin rash, combined with a slightly elevated temperature (maximum 38.2 °C), for which paracetamol (acetaminophen), ointments and cetirizine were started and alpelisib was interrupted. The dermatologist noted (generalized) urticaria covering ~30% of her body surface area (BSA), assessed as grade 2 urticaria (CTCAE v.5), with some mild edema in her lips. Altogether the reaction was assessed as drug reaction with urticaria related to alpelisib (Figure 2). Subsequently, prednisone was started 10 mg QD and increased to 20 mg QD the next day.

Together with the patient and dermatologist we discussed and decided to re-initiate alpelisib with prednisone and cetirizine as pre-medication. The rechallenge was initiated 10 days after the start of the initial reaction with almost complete resolution of the urticarial lesions (CTCAE v5 grade ≤1, <10% BSA). Approximately 1 hour after she ingested a full dose of alpelisib (300 mg), she felt tingles in her face and ears, developed redness/erythema of the skin and experienced a feeling she was running a fever, although her temperature was still 37.9 °C, all without hemodynamic irregularities. Given the mild skin rash, a second in-hospital observed rechallenge was performed with additional premedication (prednisone 40 mg QD, paracetamol 1000 mg q8hr, and cetirizine 10 mg q6hr). Ninety minutes after she took a reduced dose (150 mg) of alpelisib, she developed a type-1 allergic reaction with angioedema with a (mildly) swollen face, tingles in hands and feet, reappearance of skin rash, without hemodynamic irregularities, without signs of airway edema/obstruction or abnormalities in her laboratory parameters (Table 1). In the following hours, the skin rash aggravated. Symptomatic treatment was directly initiated (clemastine and additional hydrocortisone were given, adrenaline and IV fluids were considered but not given as no hypotension, airway edema or obstruction were present) and alpelisib was permanently discontinued. At follow-up, she had recovered from this reaction and prednisone was tapered and stopped. As a next line of treatment, she started tegafur/gimeracil/oteracil (Teysuno).

## Discussion

Alpelisib is the first oral inhibitor to selectively target the class-I phosphoinositide 3-kinase (PI3K) α-isoform and thereby block the (PI3K) pathway.^6^ In breast cancer, 35% of tumors harbor a *PIK3CA* mutation,^7^ and in specific subtypes (ie luminal A) this is up to almost 50%.^8^ Outside breast cancer, the PI3K pathway is frequently altered in endometrial (35–53%), cervical squamous cell (27%), bladder (20–23%), and colorectal cancer (15–21%). These alterations include mutations and/or amplifications of one of the genes encoding one of the four catalytic subunits (PI3Kα, PI3Kβ, PI3Kγ, and PI3Kδ).^9,10^ The PIK3CA alterations induce a transformed phenotype resulting in independent cell growth and therapy resistance.^9,10^ Although alpelisib is now only registered and approved for HR+/HER2-negative *PIK3CA*-mutated metastatic breast cancer, the prevalence of actionable PIK3CA mutations in various tumor types offers the potential to be used in many more patients, beyond its registration and approval label.

Although effective, ADRs/AEs of alpelisib are common and frequently lead to discontinuation. In the SOLAR-1 trial, a randomized phase III trial evaluating effectiveness of alpelisib with fulvestrant compared to placebo with fulvestrant for HR+/HER2-negative advanced breast cancer, 25% of patients discontinued treatment because of ADRs.^2^ The most frequent AEs leading to discontinuation were hyperglycemia (6.3%) and rash (3.2%).^2^ In the NEO-ORB trial, a randomized phase-II trial evaluating effectiveness of neoadjuvant alpelisib with letrozole compared to placebo with letrozole for HR+/HER2-negative breast cancer, 28.5% of patients on alpelisib discontinued treatment because of AEs/ADRs.^5^ Here, even higher rates of discontinuation were attributed to hyperglycemia 9% and rash 5% as most common AEs leading to discontinuation.^5^

### Incidence of Hyperglycemia and Rash

Besides being the most frequent AEs leading to discontinuation, hyperglycemia and rash were in both the NEO-ORB and SOLAR-1 trial the most common grade ≥3 adverse events. In the SOLAR-1 trial, hyperglycemia was reported in 63.7% of patients for any grade and 36.6% grade ≥3, and rash in 53.9% for any-grade and in 20.1% grade ≥3 (2). In the NEO-ORB, hyperglycemia was reported in 53.8% of patients for any grade and 26.9% grade ≥3, rash in 44.6% for any grade and in 13.3% grade ≥3 and maculopapular rash specifically in 16.9% for any grade and in 7.7% grade ≥3 (5).

The high incidence of serious AEs (including hyperglycemia and rash) seems a class-effect, and a common problem in the clinical development of PI3K inhibitors.^11^ In the SANDPIPER trial, a randomized phase III trial evaluating effectiveness of taselisib (inhibits PI3K isoforms -α, -γ, and -δ) with fulvestrant compared with placebo with fulvestrant for ER+/HER2-negative advanced breast cancer, 20% of patients discontinued taselisib due to AEs.^12^ Hyperglycemia was reported in 40.4% of patients for any grade and 10.8% grade ≥3, and rash was reported in 25.2% of patients for any grade and 3.8% grade ≥3. Rash and hyperglycaemia AEs had a lower incidence compared with the alpelisib trials; however, gastrointestinal AEs were reported more often for taselisib.^12^ These differences between the alpelisib and taselisib AE-profile could be attributed to the inhibitory effects on the different PI3K isoforms.^11^

### Management of Skin Rash

Given the high incidence of skin rash and hyperglycemia, the phase III SOLAR-1 trial protocol included specific instructions for the management of both hyperglycemia and skin/subcutaneous disorders (trial article Appendix S7 and S8).^2^ For skin rash, recommendations included symptomatic treatment with ointments, lubricants, topical, and systemic corticosteroids, the interruption of treatment in the case of grade 3 toxicity (as initially in our patients A and B) and permanent discontinuation in case of grade 4 toxicity.^2^ Adequate prophylactic therapy and early initiation of pre-medication is thought to prevent or overcome skin toxicity of PI3K inhibitors. A previous retrospective cohort study described that most patients could continue their oncologic treatment upon rechallenge (in the majority of patients without a dose reduction) without rash recurrence in 12/16 patients (75%).^3^

### Beyond Skin Rash: Hypersensitivity and Anaphylaxis

Next to rash, hypersensitivity reactions occurred in 15.7% of patients (grade ≥3: 1.7%) in the SOLAR-1 trial, although hypersensitivity reactions have not been reported as such in het NEO-ORB trial.^2,5^ In none of the previously described studies “anaphylaxis” is mentioned in either the full text or the supplements,^2,5,12^ although in these studies AEs were only reported if incidences transcended a threshold (≥10% any grade NEO-ORB; ≥10% any grade or ≥1% grade ≥3 in SANDPIPER ≥15% any grade and all SAE’s in the SOLAR-1).^2,3,5,12^ Moreover, we found no (case-)reports of anaphylactic reactions to alpelisib or taselisib in the medical literature. Still, in the summary of product characteristics (SmPC) of alpelisib, anaphylaxis is reported to have occurred during clinical trials and is categorized as “hypersensitivity,” which is reported with a prevalence of 3.9% any grade (grade 3 0.7%).^4^

Another severe hypersensitivity reaction, drug rash with eosinophilia and systemic symptoms (DRESS) has previously been reported.^4,13^ In both our cases, we found no eosinophilia, lymphocytosis, thrombocytopenia, or significant liver/kidney test abnormalities to support a diagnosis of DRESS, and based on the clinical findings, a type-I allergic (anaphylactic) reaction was considered most likely. After *any* serious hypersensitivity reaction (such as both rechallenges in cases A and B) alpelisib should be discontinued and not re-introduced, according to the SmPC/label.^4^

### Recognizing a Type-I Allergic/Anaphylactic Reaction

Although an uncommon (serious) adverse event, distinguishing a type-I allergic/anaphylactic reaction from a general and common alpelisib-associated skin rash is of vital importance. A classical type-I anaphylactic reaction is a response to prior sensitization or cross-reactivity.^14^ Prior sensitization could have happened asymptomatic or with a milder reaction (as in these cases). A serum tryptase can confirm a recent anaphylaxis event, as marker of mast cell activation, although anaphylaxis is a clinical diagnosis.^15^ In general, tryptase can be detected in serum within minutes but will normalize within 6-24 hours after the reaction.^15^ In our first case, tryptase was not elevated; however, it was unfortunately only measured after 43 h and therefore unreliable. Moreover, for the patient in case B, tryptase levels were not obtained, being a limitation to this report.

## Conclusion

PI3K inhibitors, such as PI3Kα inhibitor alpelisib, are drugs with a significant toxicity profile, including hyperglycemia and rash as common grade ≥3 adverse events. This report underlines the importance to include a developing type-I allergic reaction in the differential diagnosis for patients who develop a rash following initiation of alpelisib, especially if additional clinical findings beyond rash are present, such as urticaria, mild facial angioedema, edema, fever, dyspnea, or tachycardia. Even if an initial reaction develops slowly after days, it could still be a type-I allergic reaction. The Alpelisib label has a warning for hypersensitivity (including anaphylactic reaction) and recommends alpelisib to be permanently discontinued and should not be re-introduced in patients with serious hypersensitivity reactions. Although a rechallenge is reported to be recommended for grade 3 rash upon resolution to grade ≤1 with a reduced dose, we contest that this strategy is harmless and generalizable. This especially in patients with symptoms or signs beyond rash, such as urticaria or facial (angio)edema which are suggestive of a hypersensitivity reaction. Thus, a rechallenge could be dangerous and should always be well contemplated or even avoided if the suspicion of a type-I allergic reaction is high.

## Data Availability

The data underlying this article will be shared on reasonable request to the corresponding author.
